# A Continuous Fiber-Reinforced Additive Manufacturing Processing Based on PET Fiber and PLA

**DOI:** 10.3390/ma13143044

**Published:** 2020-07-08

**Authors:** Yuan Yao, Meng Li, Maximilian Lackner, Lammer Herfried

**Affiliations:** 1 Rapid Manufacturing Engineering Center, Mechatronic Engineering and Automation of Shanghai University, Shanghai 200444, China; smyvszhgl@i.shu.edu.cn; 2 Shanghai Key Laboratory of Intelligent Manufacturing and Robotics, Shanghai University, Shanghai 200072, China; 3 Competence Center Digital Manufacturing and Robotics, University of Applied Science Technikum Wien, Höchstädtplatz 6, Wien 1200, Austria; aburaia@technikum-wien.at; 4 Kompetenzztentrum Holz Gmbh, Altenberger Straße 69, Linz 4040, Austria; h.lammer@wood-kplus.at

**Keywords:** additive manufacture, fiber-reinforced manufacturing, continuous fiber-reinforced thermoplastic composites

## Abstract

Continuous fiber-reinforced manufacturing has many advantages, but the fabrication cost is high and its process is difficult to control. This paper presents a method for printing fiber-reinforced composite on the common fused filament fabrication (FFF) platform. Polylactic Acid (PLA) and Polyethylene terephthalate (PET) fibers are used as printing materials. A spatial continuous toolpath planning strategy is employed to reduce the workload of post-processing without cutting the fiber. Experimental results show that this process not only enables the printing of models with complex geometric shapes but also supports material recycling and reuse. A material recovery rate of 100% for continuous PET fiber and 83% for PLA were achieved for a better environmental impact. Mechanical tests show that the maximum tensile strength of continuous PET fiber-reinforced thermoplastic composites (PFRTPCs) is increased by 117.8% when compared to polyamide-66 (PA66).

## 1. Introduction

In many industrial applications, the polymer-based additive manufacturing (AM) process is mainly used to produce prototypes for design rather than functional products [1,2,3]. The main reason is that the mechanical properties of the products fabricated by current AM machines cannot reach the required level. To solve this problem, different short fibers are selected and mixed into thermoplastic matrices and printed together [4]. There are many inconsistent results reported in the literature. Some indicate that the addition of short fibers enhances the mechanical properties of pure polymers, while other experiments show that the addition of short fibers weakens the connection between the polymers. Overall, most short fiber-reinforced 3D prints have weaker performance than traditional mold-based fiber-reinforced composites. Therefore, to achieve higher performance composites, 3D printing equipment for continuous fiber-reinforced thermoplastic composites (FRTPCs) has emerged [5]. In printing products, the matrix material can act as glue, bonding the long fibers together, and protecting the continuous fibers from corrosion, degradation, and abrasion. Usually, the matrix material is subjected to the compression load, and the continuous fibers serve as the main body to bear the tensile load. In recent years, the FRTPCs have been rapidly developing, which is known as a major reinforced method for expanding the application of additive manufacturing.

The typical reinforced material used in FRTPCs is the carbon fiber. It has many advanced properties, such as the tensile strength and bending resistance close to aluminum alloy as well as lightweight and corrosion resistance. The commercial fused filament fabrication (FFF) printer that supported continuous carbon fiber-reinforced thermoplastic composites (CFRTPCs) was first developed by Mark Forged in 2014. However, due to the characteristics of carbon fiber, sharp features cannot be printed directly, and a dual extrusion head system and a cut device need to be equipped. These additional actuators increase the complexity of the machine and make the printing cost far exceed the conventional FFF platform.

In addition to carbon fibers, there are many other types of fibers that can be used as reinforcement [6,7,8,9]. In this paper, we choose to employ PET (Polyethylene terephthalate) fiber-reinforced thermoplastic composites (PFRTPCs) due to their numerous advantages. First, PFRTPCs have excellent flexibility and can be used to print surfaces with sharp features. Second, the diameter of PFRTPCs is much smaller than that of CFRTPCs, which can reduce the layer thickness and improve the printing accuracy. Third, PET fibers can provide stronger surface adhesion. Lastly, PET fibers are widely used. In the preceding century, the PET-related productions and applications have a mature industrial foundation, and the realization cost is lower. Therefore, using PET fiber as reinforcement material can meet the requirements of mechanical properties in various application scenarios, can save costs, and can facilitate recycling.

This work focuses on the three-dimensional printing of PFRTPCs based on the existing FFF platform. This is achieved by first using the prepreg method for the preparation of PFRTPCs, which avoids the in-nozzle impregnation and reduces the nonuniformity of the matrix material around the fiber. Second, a global path planning method is designed, which minimizes the post-processing workload while ensuring the continuity of the tool path. The realization of the process can simplify the printing of continuous PET fiber-reinforced composites and facilitates the recycling of continuous fibers.

Many factors in the preparation of materials and the process will affect the mechanical properties, surface quality, and processing costs of FRTPCs-based 3D printed products. Recently, there has been a flourishing of works in continuous FRTPCs on changing matrices or reinforcing materials to improve strength and stiffness of fabrication [10,11,12,13]. In particular, the current trend is to exploit the micro-structures of the matrix material and the reinforcement material during the printing and find ways to improve the mechanical properties [14]. Considering that the literature in this field is increasing rapidly, the authors only review the content related to process and path planning.

Some researchers are focused on the effect of printing FRTPCs from the perspective of materials. Matsuzaki et al. [15] demonstrated that continuous fiber reinforcement improved the tensile strength of the conventionally-printed part compared with 3D-printed polymer-based composites. Dickson et al. [1] evaluated the performance of continuous carbon, Kevlar, and glass fiber-reinforced composites manufactured using FFF, whose mechanical performances were evaluated both in tension and flexure. In order to elucidate the effects of FFF parameters on the mechanical properties of CFRTPCs, Ning et al. [16] added a different length of carbon fibers and used different fill patterns to investigate changing the mechanical properties of FFF fabricated parts. Caminero et al. [17] evaluated the effect of layer thickness and fiber volume fraction on the interlaminar bonding performance of 3D printed continuous carbon, glass, and Kevlar fiber-reinforced nylon composites.

In-nozzle printing is a common way in current FRTPCs-based 3D printing, which extrudes the matrix material and the reinforcing fiber synchronously when printing [18,19,20]. This increases the complexity of the printing platform. Due to the pull force of continuous fibers during printing, it is easy to cause the uneven distribution of matrix materials on the surface of reinforced fibers.

Path planning is used in 3D printing to generate a predefined trajectory to drive printer deposit or consolidate the material layer-by-layer. In continuous fiber-reinforced printing, the path planning will also affect the surface roughness, part strength, and manufacturability. A traditional 2D fill pattern, such as the Zigzag curve [21] and the Hilbert curve [22], can also be employed as optional approaches because they can guarantee continuity of the path. In the work from Tian et al. [23], the Zigzag fill pattern is utilized for recycling of continuous CFRTPCs. Ding et al. [24] and Li et al. [25] combine Zigzag and contour pattern strategies to generate a continuous tool path, which showed better surface accuracy. Dickson et al. [26] present a weft-warp-like woven structure, which is fabricated by a continuous path generated by scripting. The continuous grid and honeycomb fill pattern are used by Hao et al. [27] to print hollow structures with carbon fiber-reinforced thermosetting composites. A novel continuous tool path is proposed by Pattinson et al. [28] to manufacture medical devices, which match the complex form and mechanics of individual human bodies. A common problem is that there is no further discussion on the adaptability of these methods for printing structures with complex geometry.

Compared with two traditional continuous paths mentioned above, the spiral is the other choice. It can reduce sharp turns in the path, which is more suitable for generating fill patterns for printing FRTPCs. Zhao et al. [29] present a 2D continuous path planning method that uses connected Fermat spirals as the fill pattern, which is available for different complex section shapes. However, few articles were found regarding how to print complex structures with FRTPCs.

## 2. Materials and Methods

**Figure 1:**
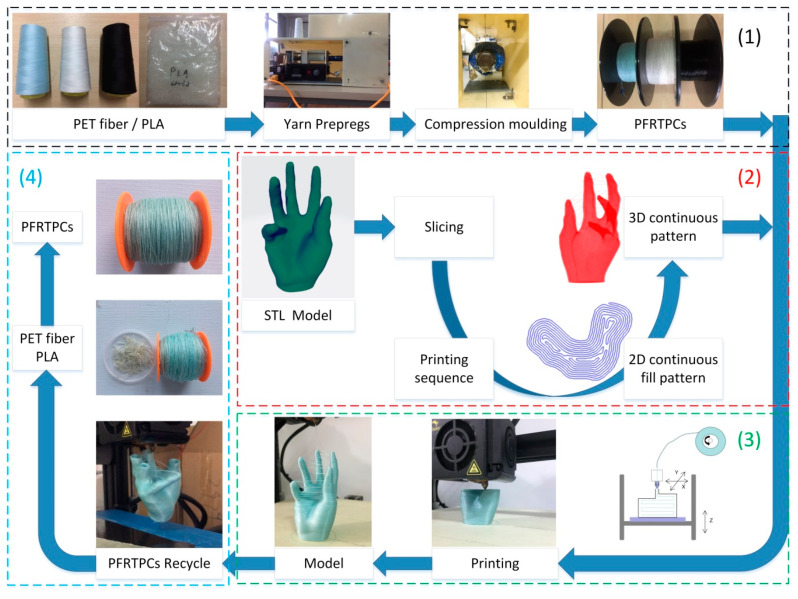
Continuous Polyethylene terephthalate (PET) fiber-reinforced printing framework.

In order to realize the printing and recycling of PFRTPCs on a common FFF platform, the process contains the preparation of materials, planning of continuous tool path, 3D printing process, materials recycling, and remanufacturing. The total PFRTPCs-based 3D printing process is shown in Figure 1.

### 2.1. Material Preparation

As discussed above, the PET fiber is deployed as a reinforcing material, which is a common type of polyester used commercially. PET is a kind of linear high-molecular weight polymer prepared by a condensation reaction. It has excellent air permeability and moisture wicking, is easy to dye, and has a strong resistance to acid, alkali, and ultraviolet (UV). Compared with carbon, PET has better light resistance. It has a strong absorption band only in the 315 nm light wave region, the strength is only lost 60% after 600 h of sunlight that is similar to cotton, and has better anti-aging ability than carbon fiber. Compared with nylon, it has better flexibility. The softening point of PET is 230–240 °C, the melting point is 255–265 °C, and the decomposition point is about 300 °C [30].

The matrix material adopted in the process is polylactic acid (PLA), which is also a commonly used thermoplastic material in FFF. The melting point of PLA is 170–230 °C, which is lower than the melting point of PET. In addition, PLA is bio-based and biodegradable, and it also has the characteristics of UV resistance.

In the process of fabrication of PFRTPCs, granular pure PLA is fed into and allowed to melt in the nozzle of a drawbench, which forms a melting pool into which the continuous PET fiber was drawn and impregnated with a melt matrix.

The diameter of the PET fiber used in this process is 0.3 mm. After prepregs, the diameter of PFRTPCs is 0.4 mm. The volume ratio of the reinforcement to the matrix is 9:7 and the mass ratio is 1:1. The common commercial PET fiber has a lower diameter, better flexibility, and a smoother surface. It is easy to carry out multiple prepregs, which can reduce the bubbles between the reinforcement material and the matrix material, and obtain the PFRTPCs with a uniform size.

### 2.2. Platform and Processing

**Figure 2:**
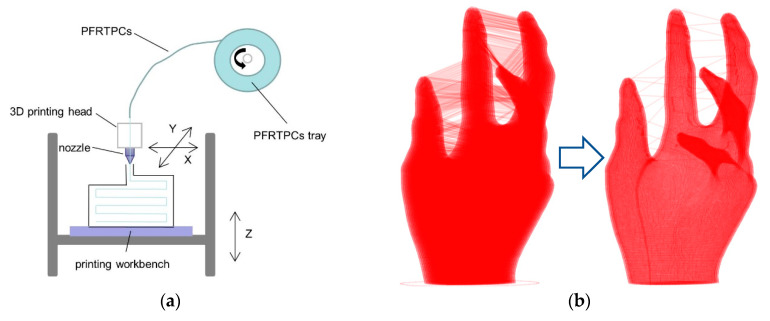
PET fiber-reinforced thermoplastic composites (PFRTPCs) printing schematic diagram. (**a**) Platform diagram. (**b**) From common infill path to a global continuous fill pattern.

Figure 2a shows the printing schematic of the PFRTPCs platform. During fabrication, the printing nozzle and the bed are heated to the set temperatures respectively, and the PFRTPCs are bonded to the heated bed through the printing nozzle. Since PFRTPCs are very soft and delicate, it is impossible to use drive gears and grooved bearings to transport materials. The printing process starts from the origin, and the fiber is pulled out of the heated nozzle by the adhesive force between the heated bed and the PFRTPCs. Then, the part can be built layer-by-layer by depositing the PFRTPCs.

#### 2.2.1. Continuous Fill Pattern

In addition, there is no cutting device on the common FFF platform. PFRTPCs have been continuously extruded throughout the forming process. All gaps between the structures on the final product will be filled with continuous fibers, as shown in Figure 2b on the left. This situation must be avoided. The solution is that the printing process is not to deposit the material on a complete layer and then print the next layer. Once the printing process determines that there is no collision between the workpiece and the nozzle, the subsequent part connected to the volume of the currently printed position will be printed first. It will then return to the lower layer to continue printing. The optimized continuous fill pattern (CFP) is shown in Figure 2b on the right side.

Assuming that the printing direction has been determined, if there is only one simply connected region in each sliced layer, it is only necessary to fill material into all regions from the bottom to the top in order. Otherwise, a traverse strategy used to guarantee the areas that are connected and do not interfere with each other can be printed first.

Formally, the input triangle mesh is sliced into a series of layers ***L*** = (*l*_1_, *l*_2_, …, *l*_n_). Each layer *l*_k_ consists of one or more simply connected regions *r*_i,k_. Then, we get a collection ***D*** = {*r*_k,i_} that includes all 2D simply connected regions layer-by-layer. Where *i* is the index of the simply connected region in each layer, *k* represents the layer index. To avoid filling, many redundant fibers in the structural gap of the work piece, the strategy adopted here is to connect the adjacent, simply connected region in the upper and lower layers, and then connect other simply connected regions, according to the geometric interference relationship between the extruder nozzle and the workpiece. The implementation of this method is based on the algorithm framework provided by Sun et al. [31]. The collision check between the extruder and printing part on the z direction is added in the process of interference. The same continuous generation is used to connect a different fill path together to a spatial continuous fill pattern for FFF.

#### 2.2.2. Processing Configurations

Based on this process, a series of experiments were performed on the common desktop FFF platform to determine a suitable set of process parameters, which are given in Table 1. In the printing stage, the diameter of the printing nozzle, layer thickness, printing temperature, printing speed, and heated bed temperature all have a direct impact on forming quality. Once the diameter of the nozzle is less than 1.5 times of the diameter of the prepreg long fiber, the viscoelastic PLA cannot be extruded with PET fibers completely, which results in nozzle blockage. The selected diameter of the nozzle is 0.6 mm, which can avoid the PLA accumulated at the nozzle during the fabrication. In addition, a layer thickness of less than 0.1 mm will also prevent the matrix material from being extruded, which will further tear the reinforcing fibers. Therefore, the layer thickness on this platform is set to 0.2 mm.

Temperature is another important factor. Considering the PLA matrix on the surface of PET fibers begins to melt before reaching the bottom of the nozzle, and accumulates over the nozzle, which will prevent the feed of PFRTPCs. On the other hand, if the set temperature is too low, the PLA does not melt completely and cannot stick to the workpiece very well. In the print stage, the temperature of the nozzle is set to 190 °C. Both the material feed and the adhesion between layers can be guaranteed. The heated bed temperature is 50 °C, which is the same as that of pure PLA.

Printing speed is a key parameter that affects printing cost, surface quality, and strength. Since no feeding mechanism is used, too high of a speed will reduce the binding strength between the two layers. The value of printing speed selected by this process is 300 mm/min. This speed range can keep the feeding and printing device running smoothly for a long time.

### 2.3. Recycling and Remanufacturing

Filaments recycling and reuse can reduce carbon emissions and help reduce increasingly serious plastic pollution. Compared with the pure thermoplastic materials, PFRTPCs is more convenient to be recycled and remanufactured after the first printing. Considering that the recycling is the reverse process of fabrication, the recycling process can still be done on the desktop FFF platform. For a product printed by continuous PFRTPCs, the recycling process starts from extracting the tail of the composite filaments from the model and pass through the extrusion head in the opposite direction. Then, the feeder retracts and collects the reinforced fiber on the roller. At this time, the matrix material melts at the place where the model contacts with the heated nozzle and can be peeled from the surface of the fiber by the nozzle. The melt PLA is fallen into the material slot on the drawbench, while the PET fiber can be rolled back onto the material stick at a constant speed. After materialize recycling, the second prepregs of yards can be done again to produce PFRTPCs for the remanufacturing process.

## 3. Results

The authors investigate the feasibility of the PFRTPCs-based process with a continuous printing path. The mechanical properties and the recyclability of materials of this process are also evaluated.

### 3.1. Process Capability Test

To verify the universality and quality of the PFRTPCs-based process, we printed different structures and evaluated the manufacturability, surface quality, and the feasibility of post-processing.

#### 3.1.1. Surface Quality

**Figure 3:**
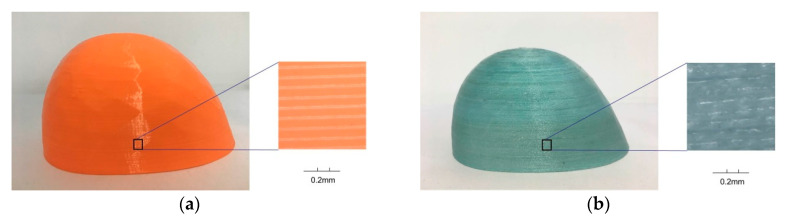
Surface of safety shoes cover. (**a**) Pure Polylactic Acid (PLA). (**b**) PET fiber-reinforced thermoplastic composites (PFRTPCs).

The object shown in Figure 3 is a safety cover, which is used to prevent heavy objects from injuring the feet of workers. It is a simple structure without the requirement of additional support during the fabrication. Once the model is sliced into a series of layers, each layer contains only one simply connected region that can be filled with a Fermat spiral-based continuous path. These continuous path segments are connected by the order of the printing sequence to form a global continuous tool path to drive the nozzle of the 3D printer to move and print the part with a continuous wire of PFRTPCs. The size of this safety shoes cover is 88.71 × 75.57 × 51.31 mm^3^. The height of each sliced layer is 0.2 mm and printing speed is 300 mm/min. In the desktop FFF platform, it takes 2 h and 53 min to print with the pure PLA. When using PFRTPCs-based printing, continuous material transport is needed. Therefore, the printing speed must be reduced. It takes 16 h and 9 min with the same platform using PFRTPCs.

The surface quality of the safety shoe cover with PFRTPCs (Figure 3b) is similar to that of safety shoe cover with PLA (Figure 3a). Shooting with an HD camera (Uhdvision 4K smart camera 3840 × 2160 @ 25 fps), there are clear and even gaps between every two layers of objects printed with PLA shown in Figure 3a,b shows the surface details of the safety shoe cover with PFRTPCs. Compared with the left, the spacing between the two deposit layers is better filled.

#### 3.1.2. Continuous Path Optimization

**Figure 4:**
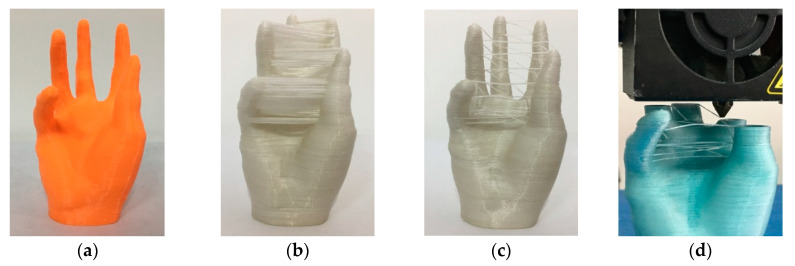
Multiple branches structure printing. (**a**) PLA with a normal path. (**b**) PFRTPCs with a normal path. (**c**) PFRTPCs with an optimized path. (**d**) Printer nozzle travels across open space.

The hand is a typical structure with multiple branches of which the size is 44.73 × 46.30 × 76.40 mm^3^. Figure 4a shows the model fabricated by a 3D printer with pure PLA, which spent about 2 h and 57 min. Figure 4b demonstrates a hand model printed with PFRTPCs while adopting continuous path filling layer-by-layer. It takes about 7 h and 16 min to print, which not only wastes PFRTPCs but also increases the difficulty of post-processing.

The surface quality of the model hand is not smooth due to the extra silk thread that cannot be removed perfectly. The print part shown in Figure 4c uses the optimized path generated by our method. The printing time is 6 h and 27 min. Compared with the traditional method, the PFRTPCs based 3D printing process not only saves printing materials but also improves printing efficiency. In addition to the advantages mentioned above, the workload of post-processing is reduced and the surface quality of the object is improved.

#### 3.1.3. Changing Materials

**Figure 5:**
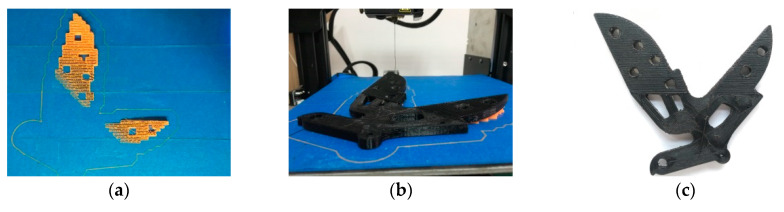
Printing of model with support structures. (**a**) Support structures with pure PLA. (**b**) Printing of bracket of bicycle rear wheel. (**c**) Product with PFRTPCs.

For the printing of objects to be supported, it is necessary to print the supporting materials (Figure 5a) before printing the objects (Figure 5b). There are two kinds of materials for the supporting structure, which includes PLA and PFRTPCs. Considering that the printing speed of PLA can reach 2000 mm/min, we select PLA as the supporting material. The best way to print the supporting structure is grid filling, which can save printing time and facilitate post-processing.

Since PLA and PFRTPCs are printed separately, it is essential to consider how to avoid interference. We generate paths for supporting the structure and model, respectively, and manually adjust the position of the object on the heated bed (Figure 5c). The sample we used in this case is a bracket of a bicycle’s rear wheel in which size is 146.11 × 135.94 × 15.07 mm^3^. The printing speed of the bracket of the bicycle real wheel is 300 mm/min and the printing time is 14 h and 19 min.

### 3.2. Mechanical Testing

To determine the mechanical strength of the PFRTPC-based printing, we carried out tensile and bending tests and compared the results with different groups of samples. The samples, the corresponding printing parameters, and test standards are listed in Table 2.

The test samples are divided into two classes. The first is composed of pure thermoplastics that contains PLA, PA66, polyethylene terephthalate glycol (PETG), and acrylonitrile butadiene styrene (ABS). The second is a fiber-reinforced class made of PFRTPCs and flax fiber-reinforced thermoplastic composites (FFRTPCs), respectively. There are five samples in each of the groups.

All of the test samples were fabricated by a customized Creality Ender-3S desktop 3D printer. Tensile strength and flexural strength were measured using a universal testing machine (TY8000, Yangzhou, China). The long PET fiber used in this experiment is the sewing machine thread QUR 13-18 produced by the HOTON GROUP Company (Yiwu, China). It contains three strands of yarn and each is composed of 40 fine fibers. The specification is 40S/3-2350Y.

**Figure 6:**
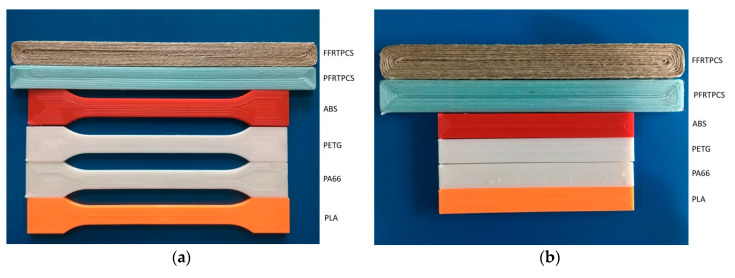
Printed test specimens. (**a**) Specimens for tensile test. (**b**) Specimens for flexural test.

The desktop 3D printer printed the tensile specimens and flexural specimens, according to printing parameters listed in Table 2. The tensile specimens and flexural specimens printed are shown in Figure 6a,b.

**Figure 7:**
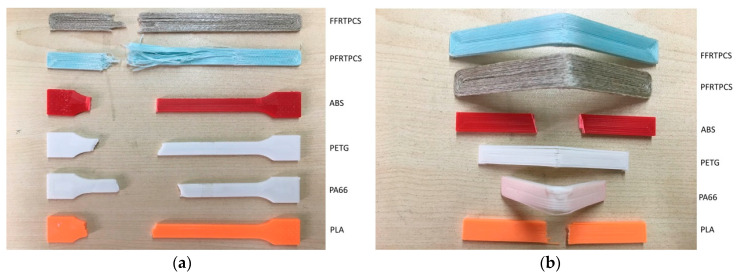
Fractured printed test specimens. (**a**) Specimens after the tensile test. (**b**) Specimens after the flexural test.

In the tensile test, the standards followed are ASTM D3039 and ASTM D638. The movement speed of the crosshead (clamping device) is 2 mm/min under a preload of 3 N. During the flexural test, the crosshead’s moving speed was 2.2 mm/min under the preload of 3 N. Figure 7 shows fractured printed test specimens.

#### 3.2.1. Tensile Test

**Figure 8:**
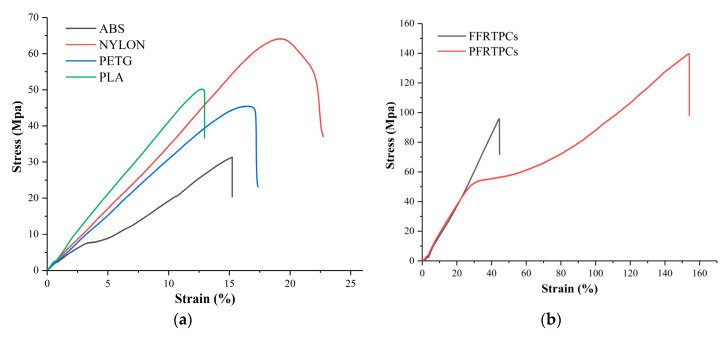
Stress-strain diagram of tensile test. (**a**) Stress-strain diagram of referenced pure polymers. (**b**) Stress-strain diagram of fiber-reinforced polymers.

Figure 8 shows the stress-strain diagrams for the tensile test. In the experiments, the stress-strain curves of the pure thermoplastic materials are similar because of the homologous properties. However, the stress-strain curves of PFRTPCs and FFRTPCs, illustrated in Figure 8b, have completely different trends because they contain different reinforced fibers.

Compared with flax fiber, PET fiber has better elongation and higher tensile strength. The direction of the stress-strain curve of PFRTPCs changed twice. At the first inflection point, the matrix PLA of PFRTPCs was pulled off. At the second inflection point, the continuous PET fiber was broken. In addition, the reinforced fiber and the matrix PLA of FFRTPCs are almost fractured at the same time.

The values of tensile strength for the test groups are shown in Table 3. The tensile strength of PFRTPCs is 139.76 MPa, while the tensile strength of FFRTPCs is 95.82 MPa. However, the maximum tensile strength of the groups made by pure thermoplastic materials is only 64.17 MPa. In addition, the tensile strength of PFRTPCs is around 46% higher than that of FFRTPCs.

#### 3.2.2. Flexural Test

**Figure 9:**
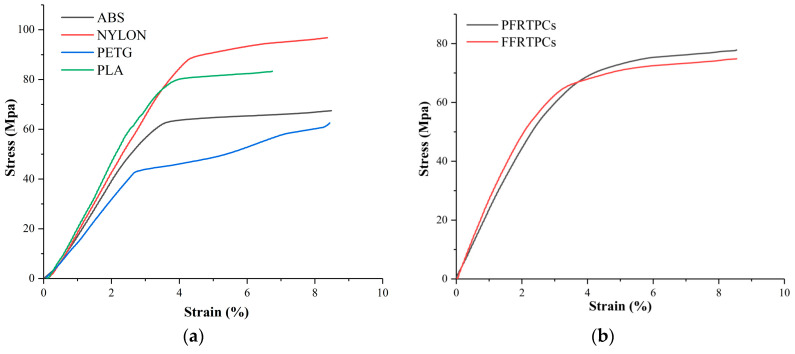
Stress-strain diagram of flexural test. (**a**) Stress-strain diagram of referenced pure polymers. (**b**) Stress-strain diagram of fiber-reinforced polymers.

In the bending test, ASTM D790 and ASTM D7079 standards are followed. Figure 9 shows the stress–strain diagrams for the flexural test. Among the four thermoplastic materials shown in Figure 9a, PA66 has the best bending performance, while PETG has the worst flexural performance. The PFRTPCs and FFRTPCs illustrated in Figure 9b have similar flexural properties.

The values of flexural strength are shown in Table 4. The flexural strength of PA66 is 98.19 MPa, which is more strength than the other five kinds of materials. The flexural strength of PFRTPCs and FFRTPCs is almost the same as that of pure PLA. The reason is that the flexural strength of fiber-reinforced thermoplastic composites mainly depends on the matrix material.

The results show that the main failure mechanisms of the printed tensile samples are fiber pull-out, fiber fracture, and delamination. Tensile samples of PFRTPCs do not break in the middle of the samples like thermoplastic materials. The place where the fracture occurs is where the clamping device is tightened. This is because the tensile sample of PFRTPCs is cut by the clamping device. Then the tensile sample is pulled. Since the outside of PFRTPC’s tensile sample is cut, it is relatively neat. However, the internal part is pulled, and the fracture surface is uneven. The bending sample of PFRTPCs is not like the sample of thermoplastic material, which is broken in the middle. The bending sample of PFRTPCs is bent. After removing the external force, the PFRTPCs bending sample can return to its original shape, but a little PLA will fall off.

### 3.3. Recycling and Remanufacturing

**Figure 10:**
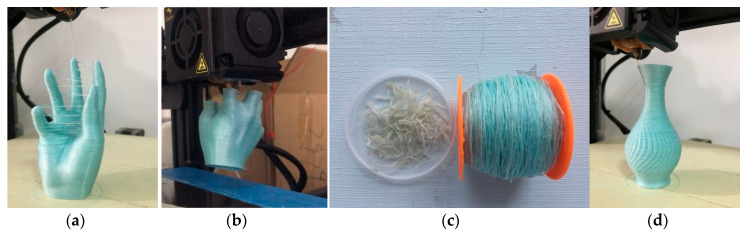
Recycling and remanufacturing. (**a**) Model printed with PFRTPCs. (**b**) Extracting materials with common FFF platform. (**c**) Recollected reinforced and matrix materials. (**d**) Remanufacturing after second prepregs.

In the experiment of recycling and remanufacturing, the same FFF platform is used to recycle the materials. During the test, the melting temperature of the drawbench is set to 200 °C, and the nozzle temperature of the 3D printer is heated to 220 °C. We found that the PLA distribution on the surface of the second prepregs fiber was more uniform than the first time. The possible reason is that more matrix material penetrates into the gaps between the fibers in the second prepregs process. The recycling process can be repeated, but because the continuous fibers are cut in post-processing, it is necessary to manually re-join the broken fibers in the recycling process. The recycling and remanufacturing process is shown in Figure 10.

The recovery of PET fiber can reach 100%, while the recovery of PLA is only 83%. The reason for the low recovery rate of PLA is that, part of the PLA covering the PET fiber is peeled off when the fiber passes through the extrusion head, but some of the debris cannot be fully collected. After remanufacturing, the tensile properties and bending properties of the remanufactured PFRTPCs samples are improved by 17% and 6%, respectively. The improvement of tensile properties is due to the fact that PLA is evenly distributed in PET fiber, while the improvement of bending properties is that PFRTPCs contains more PLA.

## 4. Discussion

Compared with the printing of pure polymers such as PLA, PA66, PETG, and ABS, PFRTPCs can double the tensile strength. The maximum tensile strength of PFRTPCs is increased by 117.8% compared to PA66. However, the maximum bending strength is reduced by 15.6% compared to PA66. The main reason is that the continuous fibers cannot increase the strength between layers. The filling path inside each layer is based on the Fermat spiral [29]. There is a lack of intersection between the layers, which will produce plenty of internal porosity. Using continuous path patterns, such as the Zigzag curve [21] and the Hilbert curve [22], alternately for each adjacent area may improve the connection strength between layers, but it will increase the complexity of the path planning algorithm. The current method needs to be further expanded to adapt to different geometries.

In terms of printing costs, PFRTPCs-based printing uses commonly used polyester fibers, and continuous fiber-reinforced printing is achieved through the common FFF platform. Compared with existing pure polymer printing, the fabrication cost depends on the cost and energy consumption of prepreg equipment. The use of continuous fibers facilitates the recycling and remanufacturing of materials. In the recycling process, because the post-processing process makes the fibers not globally continuous, the recycling process can only be designed as a semi-automated process, which requires manual assistance to find the broken fibers to continue recycling. When using recycled materials for the second prepreg, PLA can better penetrate into the polyester fiber, so the performance may be improved again. Specific evaluation in this area requires further experiments. Overall, the recycling and reuse can further reduce the manufacturing cost of this process.

## 5. Conclusions

The authors propose a continuous fiber-reinforced printing process based on a global continuous path planning method. This process uses the lowest cost PET and PLA as printing materials and expands the printing to the general FFF platform. PFRTPC-based 3D printing can enhance the mechanical strength in the layers. The bending strength and tensile strength of the printed parts are better than those of expensive materials. Furthermore, the continuous path planning simplifies the design and operation cost of the FRTPC-based 3D printing platform. This provides a feasible scheme for the development of innovative applications of long fiber-reinforced 3D printing technology.

As discussed above, this work does not enhance the adhesion between layers. The infiltration mechanism of the PET fiber and matrix materials has not been investigated. At the same time, different connected regions on the same plane are not printed continuously, and the distribution direction of long fibers under the different loads is not considered. All these reasons will affect the bonding strength between the layers and the overall performance of the workpiece. In further work, we will continue to explore the micro interaction between fibers and matrix materials in the prepreg. It is necessary to design a more automatic algorithm of continuous path planning for the model with supporting structures. The anisotropy of the workpiece can be tested and improved by planning the continuous fiber path with the interlaced or woven pattern.

## Figures and Tables

**Table 1:** Process parameters.

Diameter of Nozzle	Layer Thickness	Print Temperature	Print Speed	Heated Bed Temperature
0.6 mm	0.2 mm	190 °C	300 mm/min	50 °C

**Table 2:** The printing parameters of specimens.

Material	PLA	PA66	PETG	ABS	PFRTPCs	FFRTPCs
Diameter of material (mm)	1.75	1.75	1.75	1.75	0.4	0.8
Diameter of nozzle (mm)	0.4	0.4	0.4	0.4	0.6	1.0
Layer height (mm)	0.2	0.2	0.2	0.2	0.2	0.64
Layer numbers (tensile specimens)	16	16	16	16	16	5
Layer numbers (flexural specimens)	20	20	20	20	16	5
Tool path width (mm)	0.4	0.4	0.4	0.4	0.4	0.8
Filling percentage	100%	100%	100%	100%	100%	100%
Filling pattern	CFP	CFP	CFP	CFP	CFP	CFP
Print temperature (°C)	200	250	245	245	190	210
Heated bed temperature (°C)	50	75	80	80	50	50
Print speed (mm/min)	600	600	600	600	300	100
Print time of tensile specimens (min)	160	160	160	160	266	190
Print time of flexural specimens (min)	66	66	66	66	187	134
Tensile standard (ASTM)	D638	D638	D638	D638	D3039	D3039
Flexural standard (ASTM)	D790	D790	D790	D790	D7079	D7079

**Table 3:** Maximum load and tensile strength.

No.	Materials	Maximum Load (N)	Tensile Strength (MPa)
1	PLA	2008.26	50.21
2	PA66	2566.97	64.17
3	PETG	1818.64	45.47
4	ABS	1299.41	32.49
5	PFRTPCs	5680.83	139.76
6	FFRTPCs	5110.91	95.82

**Table 4:** Maximum load and flexural strength.

No.	Materials	Maximum Load (N)	Flexural Strength (MPa)
1	PLA	180.34	84.53
2	PA66	209.46	98.19
3	PETG	136.02	63.76
4	ABS	143.91	67.46
5	PFRTPCs	176.71	82.83
6	FFRTPCs	170.33	79.84
